# Evolutionary Paradigms from Ancient and Ongoing Conflicts between the Lentiviral Vif Protein and Mammalian APOBEC3 Enzymes

**DOI:** 10.1371/journal.ppat.1005958

**Published:** 2016-12-01

**Authors:** Reuben S. Harris, Brett D. Anderson

**Affiliations:** 1 Howard Hughes Medical Institute, University of Minnesota, Minneapolis, Minnesota, United States of America; 2 Department of Biochemistry, Molecular Biology and Biophysics, Masonic Cancer Center, Center for Genome Engineering, Institute for Molecular Virology, University of Minnesota, Minneapolis, Minnesota, United States of America; University of Kentucky, UNITED STATES

## Introduction

Lentiviruses are a unique class of retroviruses that infect a subset of mammalian species, including humans. Human immunodeficiency virus type 1 and 2 (HIV-1 and HIV-2), simian immunodeficiency virus (SIV), feline immunodeficiency virus (FIV), bovine immunodeficiency virus (BIV), maedi-visna virus (MVV), and caprine arthritis encephalitis virus (CAEV) infections result in immunodeficiency syndromes, neurological diseases, and other conditions. Disease is chronic, lifelong, and often fatal. Pathology is due in part to a capacity to immortalize by integrating into a host’s genomic DNA and a remarkable genetic plasticity that enables escape from potent adaptive immune responses without compromising vital viral functions. Lentiviruses encode three universal retroviral proteins (Gag, Pol, and Env), one lentivirus-specific protein called Vif (virus infectivity factor), and a handful of other less conserved accessory factors.

**Fig 1:**
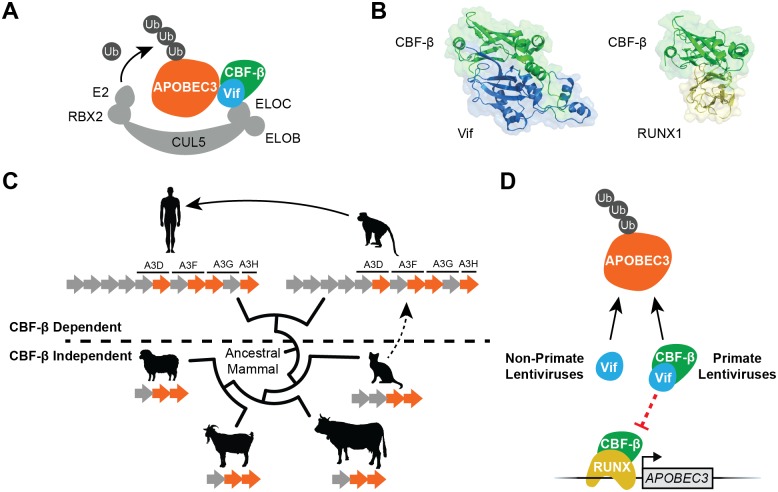
Lentiviral Vif and APOBEC3 diversity and functionality in mammals. (**A**) The primate lentiviral Vif/CBF-β complex protects viral genetic integrity by polyubiquitinating and degrading cellular APOBEC3 enzymes. (**B**) Structural representations of the Vif/CBF-β and RUNX/CBF-β heterodimeric complexes (PDB 4N9F and 1H9D, respectively). CBF-β is positioned similarly in each schematic to highlight the extensive shared interaction surface. (**C**) A schematic depicting the relative phylogenetic relationships between present-day lentivirus-infected mammals and APOBEC3 domain copy numbers (clockwise from top left, humans, non-human primates, cats, cattle, goats, and sheep; simple tree generated by Phylot). The subset of APOBEC3 domains known to interact with each species’ Vif is shaded orange. Primate lentiviruses use Vif proteins that require CBF-β, whereas non-primate lentiviruses do not (these viruses appear to lack an analogous cofactor [FIV/BIV] or to have adapted to exploit a different cellular cofactor [CypA for MVV/CAEV] [26, 27]). One-way arrows from cats to non-human primates and from non-human primates to humans depict cross-species lentivirus transmission events. (**D**) An illustration depicting the dual-functionality of primate lentiviral Vif (APOBEC3 protein degradation and *APOBEC3* gene expression inhibition) versus the mono-functionality of non-primate lentiviral Vif (APOBEC3 protein degradation).

Vif is required to protect lentiviruses from restriction by several members of the APOBEC3 family of DNA cytosine deaminases by forming a cellular polyubiquitin ligase complex to degrade these enzymes (Fig 1A). Viruses lacking Vif are inactivated because cytoplasmic APOBEC3 enzymes remain abundant, package into assembling virus particles, and prevent the production of a viable DNA copy of the viruses’ RNA genome by catalyzing the deamination of viral cDNA cytosines to uracils. Viral DNA genomes that make it through this gauntlet are rendered nonfunctional by the resulting guanine to adenine hypermutations (cDNA uracils template the insertion of genomic strand adenines that become immortalized as G-to-A mutations). In this Pearl, we review recent progress in understanding the Vif-APOBEC3 interaction and discuss a model that provides a molecular explanation for past zoonotic transmission events as well as present-day, likely ongoing optimizations that enable lentiviruses to thrive.

### CBF-β is a specific co-factor for SIV and HIV Vif function

Although HIV-1 was identified in 1983, Vif function evaded discovery for nearly 20 years, until APOBEC3G (A3G) emerged as a dominant cellular factor that strongly inhibited *vif-*deficient virus replication [1]. This advance led rapidly to elucidation of the proteasome-dependent APOBEC3 degradation mechanism, including the identification of cellular factors that Vif recruits to form the degradation complex (CUL5, ELOB, ELOC, and RBX2) (Fig 1A) [2, 3]. However, HIV-1 Vif still resisted biochemical and structural studies for nearly another decade, suggesting that the complex may be incomplete. This puzzle was solved in 2011, when the transcription cofactor CBF-β proved to be an essential part of the HIV-1 Vif ubiquitin ligase complex [4, 5]. CBF-β enabled the biochemical reconstitution of the Vif ubiquitin ligase complex [4] and, shortly thereafter, structural determination by X-ray crystallography [6]. Vif and CBF-β heterodimerize and form a scaffold for further assembly of an active ligase complex (Fig 1A and 1B).

Surprisingly, CBF-β is only essential for HIV and SIV Vif function (Fig 1C) [7]. The APOBEC3 degradation activity of non-primate lentiviral Vif proteins is unaffected by cellular CBF-β [4, 5, 8–11]. These differing genetic and biochemical requirements were initially puzzling given that all known Vif proteins function to counteract host APOBEC3 enzymes (e.g., [12]). However, the HIV/SIV Vif requirement for CBF-βmay be reconciled by postulating that this adaptation occurred when an ancestral lentivirus transmitted from a non-primate mammal into a primate. Such an event likely took place millions of years ago, because an endogenous lentivirus with a putative Vif protein exists in the present-day genomes of at least two evolutionarily diverged lemur genera [13, 14]. Because exogenously spreading lentiviruses evolve too quickly to date based on cumulative genetic diversity, endogenization events such as this effectively fossilize a virus and provide rare opportunities for estimating the age of a particular viral species and making comparisons with present day descendants such as SIV and HIV.

### CBF-β is required for *APOBEC3* gene expression and Vif likely also antagonizes this function

CBF-β normally heterodimerizes with RUNX transcription factors to regulate gene expression programs during the development of T lymphocytes (RUNX1 and RUNX3) and bone cells (RUNX2) [15]. Crystal structures of Vif/CBF-β and RUNX1/CBF-β indicate that the two complexes are mutually exclusive and suggest that competition occurs between viral Vif and cellular RUNX proteins for the same CBF-β binding surface (Fig 1B). In support, HIV-1 Vif overexpression in Jurkat T cells alters the expression of nearly 100 genes known to be regulated by RUNX1 [16]. Moreover, the expression of the HIV-restrictive *APOBEC3* genes themselves is driven by RUNX/CBF-β in CD4+ T cells (the primary reservoir for HIV-1 replication) [17]. Thus, it is likely that the binding of Vif to CBF-β also serves to deprive *APOBEC3* genes of this transcriptional activation mechanism [17]. These insights suggest that the ancestral prosimian lentiviral Vif adapted to specifically (and cleverly) hijack CBF-β in order to transcriptionally downregulate *APOBEC3* genes and thereby blunt the restrictive potential of the larger APOBEC3 protein repertoire (Fig 1C and 1D). Such an event may have created an adaptive window for optimization of the APOBEC3 protein degradation mechanism and, ultimately, provided the ancestral primate lentivirus with a two-pronged mechanism to suppress this potent antiviral defense system (Fig 1D). Subsequent spread from primate to primate and, more recently, from primates to humans may have been comparatively facile [18, 19]. It is currently difficult to know what the actual APOBEC3 repertoire was in the original lentivirus-infected prosimian species and whether its Vif function required CBF-β, but the antiviral APOBEC3 repertoire was likely complex and similar to the present-day seven-gene (11 domain) locus in all genome-annotated primates, because the bulk of the primate-specific expansion most likely took place >25 million years ago (i.e., long before lentivirus endogenization in lemurs) [20].

### Extreme heterogeneity in the interacting surface between HIV-1 Vif and different human APOBEC3 enzymes

**Fig 2:**
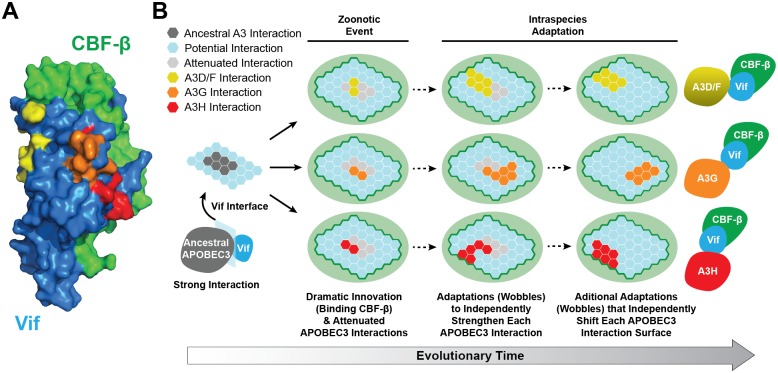
An evolutionary wobble model accounts for the dynamic nature of the interactions between the lentivirus Vif protein and host APOBEC3 enzymes. (**A**) Space-filled representation of the Vif/CBF-β complex (PDB 4N9F). Yellow, orange, and red shading highlights Vif-binding surfaces unique to A3D/F, A3G, and A3H, respectively. The A3D and A3F interaction surfaces are largely overlapping due to high levels of homology caused by a recent gene duplication. (**B**) A schematic of the “Wobble Model” for adaptation of lentiviral Vif. Left to right: The ancestral Vif-APOBEC3 interaction is predicted to be strong (illustrated by six interaction hexagons). Virus transmission to a new host with a larger APOBEC3 repertoire requires a major adaptive change, such as the binding of CBF-β during the inferred ancient lentivirus transmission from an ancestral cat to a prosimian. This event most likely reshaped Vif and attenuated the Vif-APOBEC3 interaction. However, it provided physical substrate for a series of rapid adaptations to independently strengthen each Vif-APOBEC3 interaction. The present-day, largely non-overlapping surfaces of HIV-1 Vif that interact with A3D/F, A3G, and A3H are attributable to slower and continuous adaptations driven by adaptive immune pressures.

Another remarkable feature of the interaction between HIV-1 Vif and the restrictive human APOBEC3 enzymes is that each pairwise combination maps to genetically, and likely also physically, distinct protein surfaces [21–23]. Four human APOBEC3 enzymes contribute to restricting HIV-1 infection (Fig 1C). A3G preferentially deaminates 5′CC to CU in viral cDNA and causes a genomic strand 5′GG to AG mutation bias. APOBEC3D (A3D), APOBEC3F (A3F), and APOBEC3H (A3H) preferentially deaminate 5′TC to TU in viral cDNA, which together explain genomic strand 5′GA to AA mutations. Many studies have identified amino acid substitution mutations in Vif and each APOBEC3 enzyme that specifically disrupt individual pairwise interactions and leave the others intact (for recent work, see [21–23]). For instance, separation-of-function mutations in Vif that disrupt the Vif-A3F interaction map to three distinct clusters. Different clusters of Vif amino acid substitutions specifically disrupt the interaction with A3G, and different clusters with A3H. Remarkably, upon visualization of the three-dimensional structure of the Vif-CBF-β complex, each of these linearly separated amino acid clusters combines to form largely discrete solvent-accessible interaction surfaces (Fig 2A). Likewise, A3D/F, A3G, and A3H each have a structurally distinct surface to interact with Vif.

### A wobble model explains the dynamic nature of the Vif-APOBEC3 pathogen-host interaction

Given functional conservation of the Vif-APOBEC3 interaction across species, an early assumption was that Vif would target a conserved structural element in all APOBEC3 enzymes, such as active site residues required for DNA cytosine deamination. Such a mechanism for molecular engagement would facilitate rapid virus dissemination both within and between species. However, as shown by different Vif-APOBEC3 interaction surfaces, this prediction is clearly incorrect (Fig 2A). Can an evolutionary model reconcile these structural and functional data in a manner that explains both the variation in the Vif-APOBEC3 interaction surfaces and the profound adaptive change that occurred during cross-species transmission into an ancestral primate?

A “Wobble Model” may account for these seemingly disparate and complex observations (Fig 2B) [21]. This model is grounded in the high likelihood that an ancestral lentivirus was already established in a non-primate mammal with a relatively simple APOBEC3 repertoire prior to transmitting into a primate with a more complex repertoire (Fig 1C). A provocative scenario, due to frequent blood transmission opportunities from cohabitation and predator–prey relationships, is transmission from an ancestral felid (carnivorous cat) to a prosimian. In this feasible, albeit presently hypothetical example, a felid lentiviral Vif protein optimized for the smaller cat APOBEC3 repertoire would, during the course of transmission, have had to adapt rapidly to the larger primate APOBEC3 repertoire. This cross-species “jump” was most likely facilitated by hijacking CBF-β to counteract APOBEC3-mediated restriction through the aforementioned two-pronged mechanism (Fig 1D). In support of this model, SIV Vif still has the capacity to degrade feline APOBEC3 enzymes in a CBF-β dependent manner [10, 24, 25]. Moreover, this newly transmitted virus most likely initially produced a Vif protein capable of suboptimal degradation of the APOBEC3 enzymes of its new host. Therefore, rapid adaptation and re-optimization of the Vif protein was probably required for virus replication and dissemination. This initial rapid adaptation most likely occurred in the initial host, or the virus would have succumbed to restriction and never spread throughout the species. At the molecular level, the Vif-APOBEC3 interaction was likely strengthened by a rapid expansion of the interaction interface.

Importantly, after cross-species transmission, adaptation to each of the new host’s restrictive APOBEC3 enzymes will occur independently, as each exerts its own unique selective pressure. For example, an amino acid change at one position (a wobble) may alter the interaction with one host APOBEC3 enzyme but not others. Therefore, in an iterative fashion, a single Vif-APOBEC3 interaction surface will begin to diverge from the others. Over an evolutionary time scale, fueled by adaptive immune pressure, it is easy to imagine how an ancestral Vif-APOBEC3 interaction may have undergone many stepwise wobbles to present-day configurations. One prediction of this model is the likelihood that Vif must maintain at least a partial interaction with each restrictive host APOBEC3 enzyme during the adaption period (or face extinction). This helps explain why all known Vif-APOBEC3 interactions occur on discrete solvent exposed surfaces (e.g., present day HIV-1 Vif interactions with human A3D/F, A3G, and A3H; Fig 2A). A corollary is that no pairwise combination of Vif-APOBEC3 interactions will be the same unless an *APOBEC3* gene duplication or gene conversion event creates a mimicry scenario such as for present-day human A3D and A3F.

### Summary

Recent studies on the Vif-APOBEC3 interaction have yielded many surprises, including different cofactor requirements and incredible molecular heterogeneity even within a single species, such as the interactions between HIV-1 Vif and human A3D/F, A3G, and A3H. These disparate binding interfaces and the incredible evolutionary flexibility of HIV-1 Vif do not bode well for the development of a universal Vif inhibitor to disrupt the Vif-APOBEC3 interface. Other heterologous surfaces of the ligase complex are more attractive drug targets. Most importantly, this interaction has yielded critical molecular information and a general wobble model for pathogen-host evolution with potential for broader applicability. Critical features of this model are rapid pathogen evolution and continuous adaptations (interaction wobbles) driven by adaptive immune responses within infected individuals. More extreme pathogen adaptations, such as those required for cross-species transmission of ancestral lentiviruses and neutralization of more complex repertoires of restriction factors, are mediated by rare events such as the hijacking of cellular CBF-β.
